# Early Risk Prediction Model for Stroke-Heart Syndrome Following Endovascular Therapy

**DOI:** 10.14336/AD.2025.10710

**Published:** 2026-08-01

**Authors:** Lanjing Wang, Shuangfeng Huang, Yongle Wang, Omar Elmadhoun, Changhong Ren, Wenbo Zhao, Yao Yu, Guiyou Liu, Xunming Ji, Sijie Li

**Affiliations:** ^1^Department of Neurology, Xuanwu Hospital, Capital Medical University, Beijing, China.; ^2^Division of Critical Care, Department of Anesthesiology & Perioperative Medicine, Mayo Clinic, Rochester, MN 55905, USA.; ^3^Beijing Key Laboratory of Hypoxic Conditioning Translational Medicine, Xuanwu Hospital, Capital Medical University, Beijing, China.; ^4^Beijing Institute for Brain Disorders, Capital Medical University, Beijing, China.; ^5^Neuro Cardio Vascular Diseases Center, Xuanwu Hospital, Capital Medical University, Beijing, China.; ^6^Clinical Center for Combined Heart and Brain Disease, Capital Medical University, Beijing, China

**Keywords:** Acute ischemic stroke, endovascular therapy, nomogram, risk prediction model, stroke-heart syndrome

## Abstract

Stroke-heart syndrome (SHS) significantly impacts patient prognosis, and reperfusion treatment strategies may have an impact on the occurrence of SHS following acute ischemic stroke (AIS). This study aimed to develop a nomogram-based SHS prediction model for anterior circulation stroke patients after endovascular therapy (EVT), addressing the current gap in early risk stratification of this population. This retrospective study enrolled 218 AIS patients who underwent EVT between January 2013 and June 2021, with an observed SHS incidence of 13.8% within the first two weeks post-EVT. We used the least absolute shrinkage and selection operator regression and multivariate logistic regression analysis to identify variables strongly associated with SHS. The results showed that age (OR 1.060, 95% CI 1.021-1.100, P = 0.002), hyperlipidemia (OR 3.400, 95% CI 1.289-8.968, P = 0.013), creatinine (OR 1.023, 95% CI 1.000-1.046, P = 0.049), and total anterior circulation infarct (TACI, OR 4.875, 95% CI 1.984-11.980, P = 0.001) were significantly associated with SHS and were subsequently incorporated into the construction of a nomogram-based prediction model. The area under receiver-operating characteristic curve (AUC), calibration curve, Hosmer-Lemeshow test, and Brier score were employed to comprehensively assess the accuracy and calibration of this model. The results demonstrate that the model exhibits good discriminatory ability (AUC = 0.812), calibration (Hosmer-Lemeshow test P = 0.855, Brier score = 0.098), and robustness (internal cross-validation AUC = 0.811). Furthermore, we assessed neurological outcomes at 3 months post-stroke using the modified Rankin Scale and found that SHS was independently associated with an increased risk of unfavorable functional outcome (OR 3.267, 95% CI 1.159-9.212, P = 0.025). In conclusion, SHS significantly increases the risk of unfavorable outcomes in AIS patients undergoing EVT. The nomogram, incorporating age, hyperlipidemia, TACI, and creatinine, exhibits strong predictive accuracy for early SHS; nevertheless, multicenter prospective validation is warranted prior to clinical implementation.

## INTRODUCTION

Stroke-heart syndrome (SHS) has emerged as an important clinical topic since its initial characterization by Byer et al. in 1947, with subsequent formal definition by Scheitz et al. in 2018 [1,2]. SHS encompasses a spectrum of cardiac complications after stroke, ranging from mild, asymptomatic electrocardiographic changes to severe conditions like acute myocardial infarction and life-threatening ventricular arrhythmias [2,3]. The reported incidence of SHS varies widely due to inconsistencies in observation periods and definitions. Notably, previous studies have shown that the incidence and mortality of SHS peak within the first two weeks following ischemic stroke onset [4]. Acute ischemic stroke (AIS) patients who develop SHS exhibit significantly worse clinical outcomes compared to those without cardiac complications [5,6]. For instance, poststroke heart failure increases in-hospital mortality by 2.5 times, while poststroke acute myocardial injury triples this risk [7,8].

Given SHS's significant impact on post-stroke prognosis, early risk stratification is crucial. Several studies have examined predictive factors of cardiac complications after AIS, such as advanced age, severe stroke, elevated creatinine, and insular infarction [4,9-11]. Prior research has developed a risk prediction scale incorporating age, gender, coagulation indexes, neutrophils, carotid artery stenosis, and National Institutes of Health Stroke Scale (NIHSS) score to identify high-risk SHS patients, irrespective of the treatment they received [12]. The development of context-specific predictive models with optimal parsimony is essential for improving the precision and clinical utility of SHS risk stratification.

Intravenous thrombolysis (IVT) and endovascular therapy (EVT) are the primary methods for restoring blood flow in early-stage AIS [13-15]. EVT plays a pivotal role in achieving rapid reperfusion for anterior circulation large vessel occlusion strokes, potentially demonstrating superior therapeutic effects compared to IVT alone [16-18]. A recent study found that reperfusion treatment strategies may have an impact on the occurrence of SHS after stroke [19].

Given the absence of risk prediction models tailored for EVT patients, this study aimed to identify early predictive factors of SHS and develop a nomogram-based model for anterior circulation stroke patients undergoing EVT.

## METHODS

### Study design and participants

This retrospective observational study enrolled consecutive anterior circulation AIS patients who underwent EVT between January 2013 and June 2021. The study was conducted in accordance with the 1964 Helsinki Declaration and its subsequent amendments. The Ethics Committee of Xuanwu Hospital of Capital Medical University approved the study (approval no. [2024]067-003) and granted a waiver of informed consent due to its retrospective nature.

Adult patients diagnosed with AIS and treated with EVT were screened for inclusion. Participants were excluded if they met any of the following criteria: (1) history of coronary heart disease, arrhythmia, heart failure, valvular heart disease or other clinically diagnosed cardiac conditions; (2) history of stroke; (3) etiology not classified as large artery atherosclerosis according to the Trial of Org 10 172 in Acute Stroke Treatment (TOAST) criteria; (4) posterior circulation stroke. Patients meeting the criteria were included in the statistical analysis for model development. Additionally, patients were followed up for 3 months, with those lost to follow-up excluded from the final prognostic analysis.

### Data collection

Demographic information and clinical data, including vascular risk factors, physical examination, infarction severity, infarction site, laboratory tests, and treatment-related details for each patient, were systematically collected and presented in detail in Table 1. The systemic immune-inflammation index is calculated as follows: Platelet count × neutrophil count / lymphocyte count. NIHSS score and Alberta Stroke Program Early Computed Tomography Score (ASPECTS) of each patient were evaluated by qualified neurologists. The Modified Thrombolysis in Cerebral Infarction (mTICI) scale was used to grade the recanalization outcome, with an mTICI of 2b/3 considered successful reperfusion.

### Definition of Outcomes

(1) SHS: This composite end point consisted of any of the following manifestations of secondary cardiac damage within two weeks after AIS onset: (a) acute coronary syndrome (ACS); (b) arrhythmia detected by electrocardiogram, such as atrial fibrillation, ventricular fibrillation, ventricular tachycardia, and cardiac arrest; (c) cardiac dysfunction showed by echocardiography, such as left ventricular systolic/diastolic dysfunction (with or without reduced left ventricular ejection fraction), and abnormalities in ventricular wall motion; (d) cardiogenic death [4]. Based on the occurrence of cardiac complications, patients were categorized into the SHS group or the non-SHS group.

(2) Neurofunctional prognosis: The modified Rankin Scale (mRS) score, evaluated by experienced neurologists, was used to assess the neurofunctional prognosis at three months after stroke. Patients with an mRS score exceeding 2 were deemed to have an unfavorable prognosis.

### Statistical analysis

For variables with <5% missingness, quantitative variables were imputed using median values while qualitative variables employed mode imputation. Following an evaluation of data distributions using the Kolmogorov-Smirnov test, quantitative data were presented as mean ± standard deviation for normally distributed data or median with interquartile range (IQR) for non-normally distributed data. To compare differences between the two groups, Student's t-test or Mann-Whitney U test was employed. Qualitative data was depicted as frequency and percentage, and statistical significance between groups was assessed using the Chi-square test or Fisher's exact test, depending on the expected frequencies.

First, least absolute shrinkage and selection operator (LASSO) regression analysis, coupled with 10-fold cross-validation, were performed to screen significant variables, employing λ.min as the selection criterion. The sample size met the minimum sample size required based on the number of predictor variables [20]. Subsequently, the selected variables were integrated into a multivariate logistic regression analysis. Using the statistically significant variables from this analysis, a nomogram prediction model was formulated. A receiver-operating characteristic (ROC) curve was employed to assess the discriminatory ability and accuracy of the model based on the area under ROC curve (AUC). A calibration curve, Hosmer-Lemeshow good of fit test, and Brier score were conducted to measure the model’s calibration. Decision curve analysis (DCA) was utilized to assess the net benefit of the tool in clinical application for patients. Finally, to test the robustness of the nomogram, internal validation of this regression model was operated based on 1,000 bootstrap replicates. And we additionally conducted subgroup analyses separately for patients with ACS, arrhythmia, cardiac dysfunction, and SHS excluding mild arrhythmia.

All statistical data analysis in this study were performed with the Statistical Package for the Social Sciences (SPSS) software version 26.0 (Chicago, IL, USA), R software version 4.3.2, and GraphPad Prism 8.0. A two-sided P-value < 0.05 was considered statistically significant.

**Table 1:** Characteristics of patients in the SHS group and the non-SHS group.

	Total (n=218)	SHS (n=30)	Non-SHS (n=188)	P
***Demography***				
Age, years	59.23 (11.81)	66.53 (12.80)	58.06 (11.24)	<0.001^***^
Female, n (%)	45 (20.6)	11 (36.7)	34 (18.1)	0.020^*^
BMI, kg/m^2^	25.39 (23.29-27.26)	25.86 (22.38-28.44)	25.39 (23.40-27.04)	0.928

***Vascular risk factors***				
Hypertension, n (%)	143 (65.6)	25 (83.3)	118 (62.8)	0.028^*^
Diabetes, n (%)	56 (25.7)	12 (25.7)	44 (23.4)	0.053
Hyperlipidemia, n (%)	120 (55.0)	23 (76.7)	97 (51.6)	0.010^*^
Smoke, n (%)	113 (51.8)	13 (43.3)	100 (53.2)	0.316
Alcohol, n (%)	93 (42.7)	11 (36.7)	82 (43.6)	0.475

***Physical examination***				
Admission SBP, mmHg	144.00 (130.00-159.00)	151.50 (137.00-161.00)	141.00 (129.25-157.00)	0.066
Admission DBP, mmHg	84.00 (77.75-90.00)	81.50 (71.75-90.00)	85.00 (78.00-90.00)	0.430

***Infarction severity***				
NIHSS score	14.00 (10.00-18.00)	16.50 (12.00-22.25)	14.00 (10.00-18.00)	0.022^*^
ASPECT score	9.00 (7.00-10.00)	8.50 (7.00-10.00)	9.00 (7.00-10.00)	0.957

***Infarction site***				
TACI, n (%)	43 (19.7)	14 (46.7)	29 (15.4)	<0.001^***^
Insular involvement, n (%)	113 (51.8)	19 (63.3)	94 (50.0)	0.175
Right insular	51 (23.4)	9 (30.0)	42 (22.3)	0.357
Left insular	167 (76.6)	21 (70.0)	146 (77.7)	0.357

***Laboratory tests***				
Leukocyte, ×10^9^/L	8.83 (7.13-10.92)	9.63 (8.28-10.34)	8.75 (7.01-11.18)	0.259
Neutrophil/L, ×10^9^/L	6.95 (5.10-9.08)	8.17 (6.07-8.77)	6.67 (4.97-9.39)	0.283
Lymphocyte, ×10^9^/L	1.27 (0.87-1.85)	1.18 (0.78-1.92)	1.28 (0.89-1.85)	0.677
Monocyte, ×10^9^/L	0.39 (0.31-0.50)	0.40 (0.28-0.60)	0.39 (0.31-0.50)	0.805
Erythrocyte, ×10^12^/L	4.58 (0.57)	4.40 (0.65)	4.60 (0.55)	0.065
Hemoglobin, g/L	144.00 (130.00-155.00)	134.50 (123.00-154.75)	145.00 (130.00-155.00)	0.179
Platelet, ×10^9^/L	213.00 (179.00-252.50)	233.50 (178.00-258.75)	213.00 (179.00-252.00)	0.540
SII	1150.18 (635.17-2101.69)	1509.94 (644.67-2343.36)	1078.05 (629.40-1969.33)	0.223
MPV, fl	9.90 (9.30-10.50)	9.90 (9.18-10.38)	9.90 (9.30-10.50)	0.869
Platelet crit, %	0.21 (0.18-0.25)	0.22 (0.19-0.25)	0.21 (0.18-0.25)	0.469
PDW, %	10.60 (9.60-12.10)	10.90 (9.35-12.25)	10.55 (9.60-12.08)	0.959
D-dimer, mg/L	0.71 (0.35-1.91)	1.33 (0.61-2.42)	0.69 (0.34-1.85)	0.076
Glucose, mmol/L	7.06 (6.12-8.97)	7.66 (6.57-10.65)	6.98 (6.07-8.65)	0.061
Creatinine, μmol/L	63.00 (53.00-72.00)	64.00 (50.75-77.50)	63.00 (53.00-71.00)	0.548
Urea, mmol/L	5.14 (4.02-6.41)	6.00 (5.00-7.23)	5.00 (3.90-6.21)	0.007^**^
ALT, U/L	19.00 (14.00-27.00)	18.00 (14.00-22.00)	19.00 (13.25-28.00)	0.411
AST, U/L	23.00 (20.00-30.00)	24.00 (21.00-30.50)	23.00 (20.00-30.00)	0.370
Albumin, g/L	38.71 (36.28-41.82)	38.19 (34.93-41.60)	38.77 (36.40-41.87)	0.430
Prealbumin, g/L	237.22 (62.68)	220.97 (51.00)	239.81 (64.09)	0.127
Uric acid, μmol/L	326.00 (272.50-375.00)	321.50 (282.25-376.50)	326.00 (266.00-375.00)	0.868
Cholesterol, mmol/L	4.58 (4.06-5.17)	5.03 (4.41-5.39)	4.52 (3.89-5.16)	0.061
Triglyceride, mmol/L	1.25 (0.82-2.06)	1.50 (0.79-2.27)	1.19 (0.82-1.99)	0.407
HDL, mmol/L	1.16 (0.99-1.33)	1.18 (1.08-1.32)	1.16 (0.98-1.34)	0.256
LDL, mmol/L	2.88 (2.42-3.50)	3.11 (2.67-3.65)	2.88 (2.37-3.49)	0.185
Apoprotein A, g/L	1.19 (1.10-1.32)	1.22 (1.19-1.42)	1.19 (1.08-1.32)	0.103
Apoprotein B, g/L	0.90 (0.80-1.09)	0.93 (0.84-1.15)	0.90 (0.80-1.09)	0.390

***Treatment-related information***				
Intravenous thrombolysis, n (%)	79 (36.2)	9 (30.0)	70 (37.2)	0.444
Tirofiban, n (%)	159 (72.9)	21 (70.0)	138 (73.4)	0.697
Time onset-to- puncture (min)	380.00 (297.50-540.00)	367.50 (264.75-601.25)	381.00 (300.00-528.75)	0.830
General anesthesia, n (%)	65 (29.8)	8 (26.7)	57 (30.3)	0.685
Intra-arterial thrombolysis, n (%)	5 (2.3)	1 (3.3)	4 (2.1)	0.527
Thrombus aspiration, n (%)	100 (45.9)	16 (53.3)	84 (44.7)	0.377
Stent thrombectomy, n (%)	162 (74.3)	21 (70.0)	141 (75.0)	0.561
Using balloon, n (%)	63 (28.9)	8 (26.7)	55 (29.3)	0.771
Stent implantation, n (%)	91 (41.7)	13 (43.3)	78 (41.5)	0.849
Surgery, n (%)	15 (6.9)	4 (13.3)	11 (5.9)	0.265
mTICI 2b/3, n (%)	198 (90.8)	28 (93.3)	170 (90.4)	0.864
Hemorrhagic transformation, n (%)	79 (36.2)	13 (43.3)	66 (35.1)	0.384
sICH, n (%)	23 (10.6)	4 (13.3)	19 (10.1)	0.830

Data are presented as mean (SD)/medians (IQR) and frequencies (%). SHS, stroke-heart syndrome; BMI, body mass index; SBP, systolic blood pressure; TACI, total anterior circulation infarct; SII, systemic immune-inflammation index; MPV, mean platelet volume; PDW, platelet distribution width; ALT, alanine aminotransferase; AST, aspartate aminotransferase; HDL, high-density lipoprotein cholesterol; LDL, low-density lipoprotein cholesterol; sICH, symptomatic intracranial hemorrhage, MMI, malignant middle cerebral artery infarction. * P < 0.05, ** P < 0.01, *** P < 0.001.

## RESULTS

### Overall baseline characteristics

**Figure 1.**
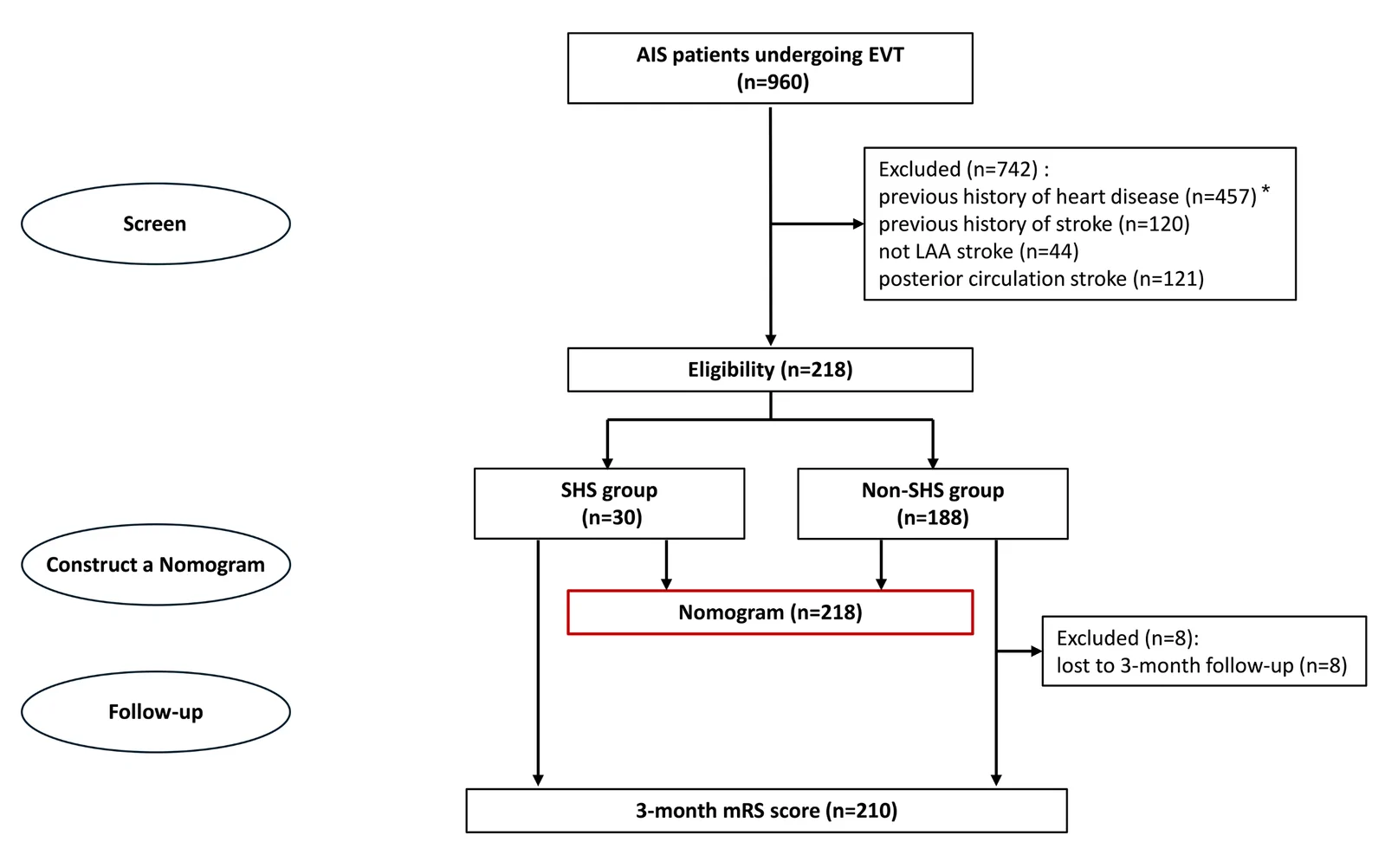
**The elaborate enrollment flow of the study**. *The study excluded 457 patients with previous heart diseases, including 236 cases of coronary artery disease, 78 cases of heart failure, 322 cases of arrhythmia, 48 cases of valvular heart disease, and 23 cases of other cardiac conditions.

Between January 2013 and June 2021, 960 AIS patients undergoing EVT were initially screened. The elaborate enrollment flow was depicted in Figure 1. According to the exclusion criteria, 742 patients were excluded. Ultimately, 218 patients were incorporated into the study, comprising 173 (79.4%) males with a mean age of 59.23 ± 11.81 years. Forty-three (19.7%) patients had total anterior circulation infarct (TACI), with an admission NIHSS score of 14.00 (IQR 10.00-18.00). Among them, 79 (36.2%) patients received IVT before EVT. One hundred (45.9%) patients underwent thrombus aspiration, while 162 (74.3%) underwent stent thrombectomy. After therapy, 198 (90.8%) patients achieved successful recanalization (Table 1).

### Incidence of cardiac complications

Among 218 patients, 30 (13.8%) patients experienced cardiac complications, including 8 cases ACS, 8 cases arrhythmia, 23 cases cardiac dysfunction, and 2 cases cardiogenic death. Compared to the non-SHS group, SHS patients were older (66.53 vs. 58.06 years, P < 0.001), more frequently female (36.7% vs. 18.1%, P = 0.020), and had higher baseline NIHSS scores (16.50 vs. 14.00, P = 0.022) and serum urea levels (6.00 vs. 5.00 mmol/L, P = 0.007). Additionally, the SHS group had higher proportions of hypertension (83.3% vs. 62.8%, P = 0.028), hyperlipidemia (76.7% vs. 51.6%, P = 0.010), and TACI (46.7% vs. 15.4%, P < 0.001).

### Prediction model construction

**Figure 2.**
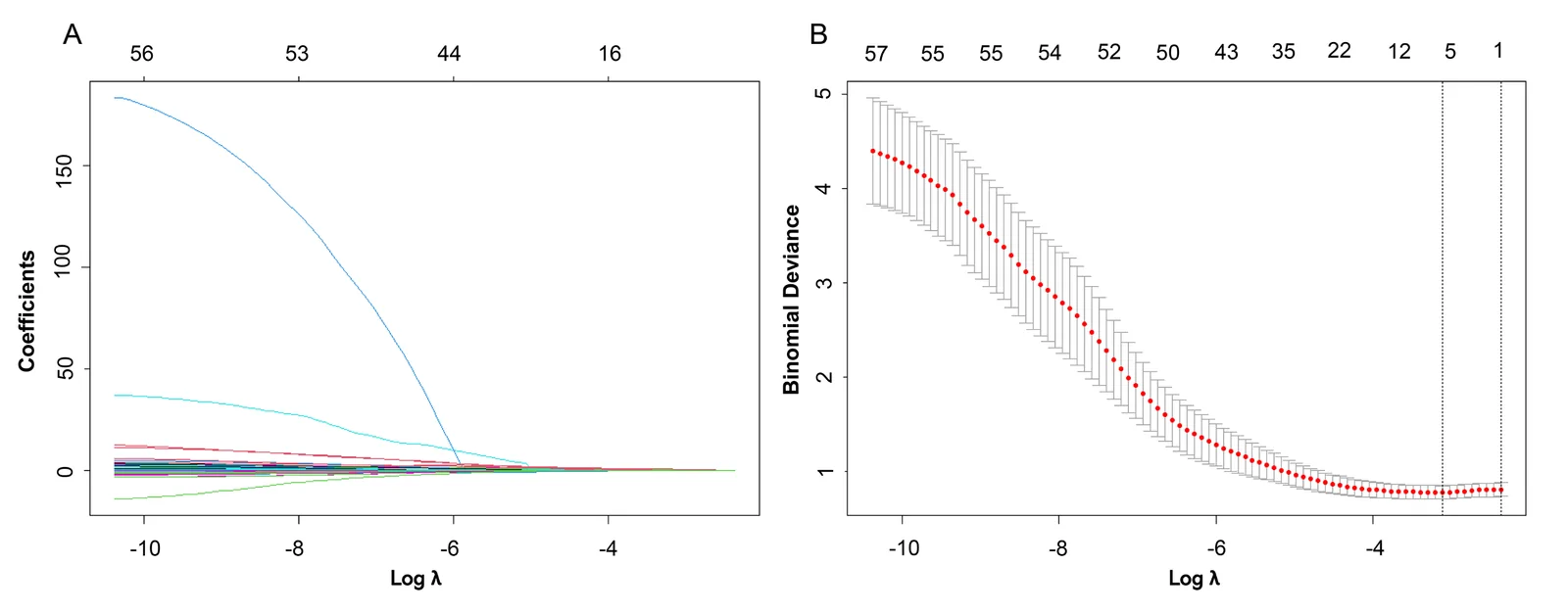
**LASSO regression for variable screening**. (**A**) LASSO coefficient path. The Lasso regression coefficient profiles of all baseline characteristics. (**B**) LASSO regularization path. The optimal λ selection in Lasso regression after 10-fold cross-validation. Two vertical dashed lines represent the optimal values under the minimum criterion and the 1-SE criterion, respectively.

**Figure 3.**
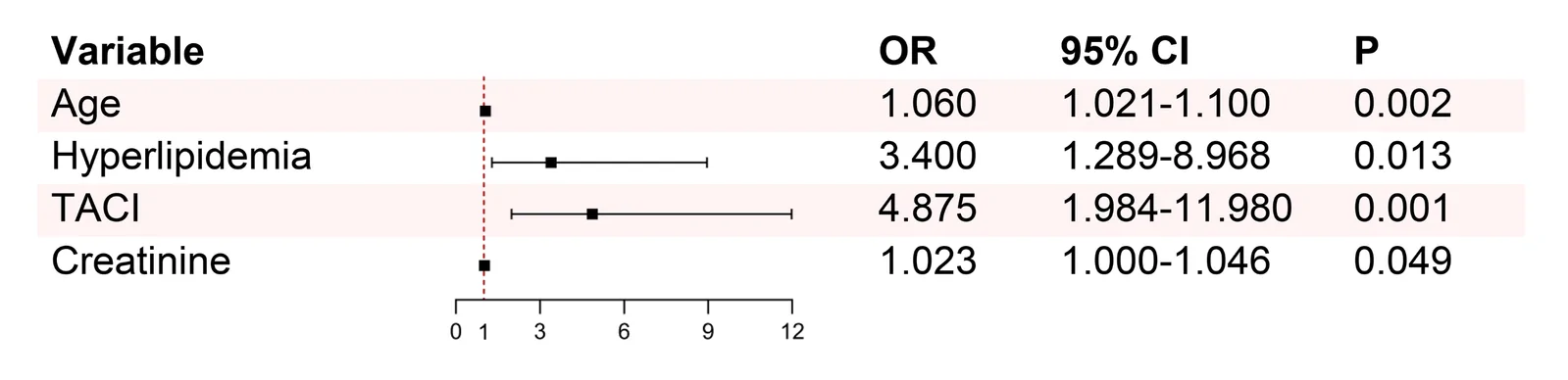
**Multivariate logistic regression analysis with stroke-heart syndrome (SHS) as dependent variable**. TACI, total anterior circulation infarct.

LASSO regression analysis was first employed to screen significant variables. Six variables strongly correlated with SHS were identified based on a 10-fold cross-validation with the λ.min of 0.044 (Fig. 2A, B). Age, diabetes, hyperlipidemia, NIHSS score, creatinine, and TACI were further included in the multivariate logistic regression analysis. The result shows that age (OR 1.060, 95% CI 1.021-1.100, P = 0.002), hyperlipidemia (OR 3.400, 95% CI 1.289-8.968, P = 0.013), creatinine (OR 1.023, 95% CI 1.000-1.046, P = 0.049), and TACI (OR 4.875, 95% CI 1.984-11.980, P = 0.001) are significantly associated with SHS in AIS patients following EVT (Fig. 3).

**Figure 4.**
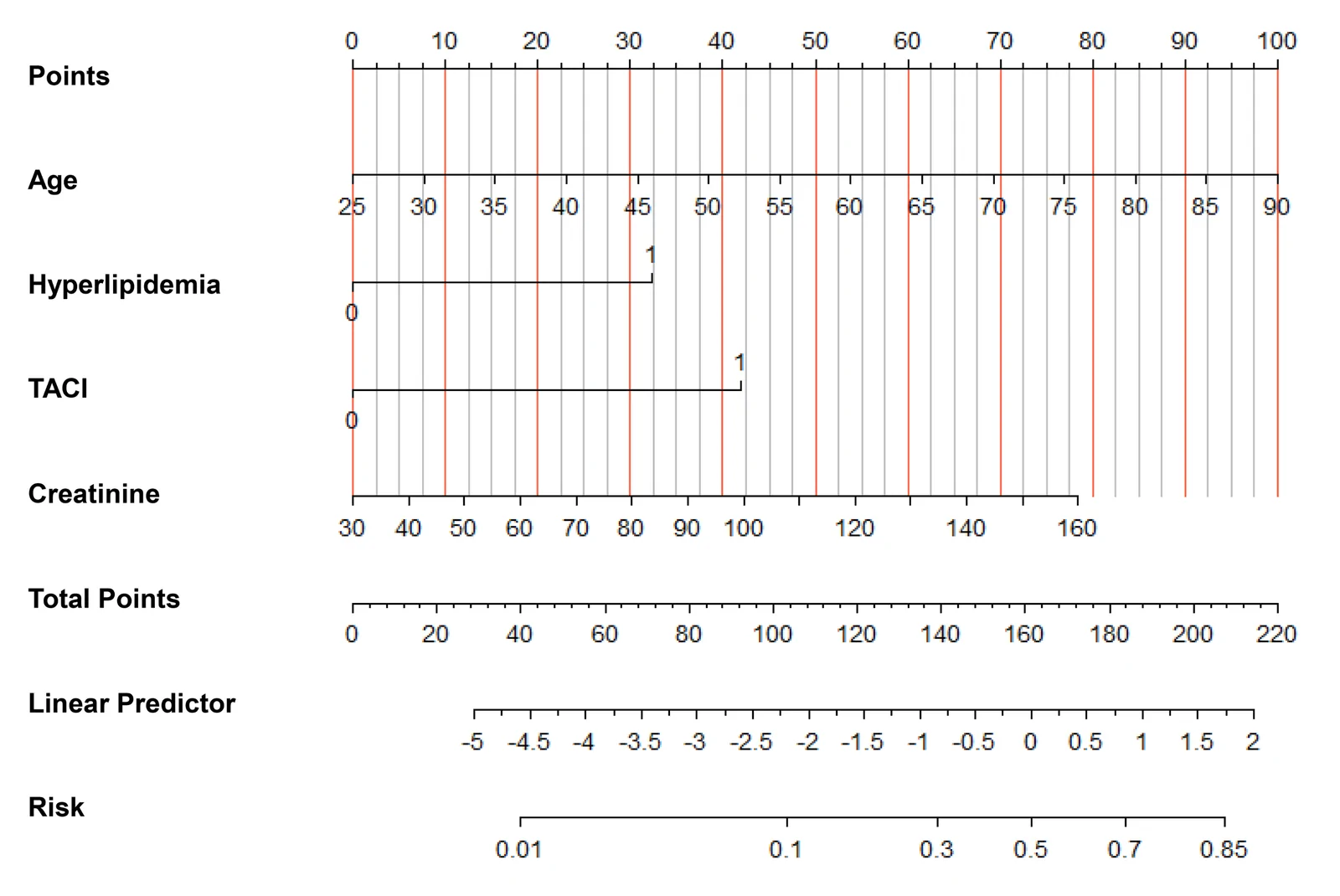
**Nomogram for SHS risk stratification**. The nomogram is constructed based on age, hyperlipidemia, creatinine, and TACI. To estimate individual SHS risk, locate patient values on each variable axis, sum the corresponding points across all predictors, and project the total points to the bottom probability scale.

**Figure 5.**
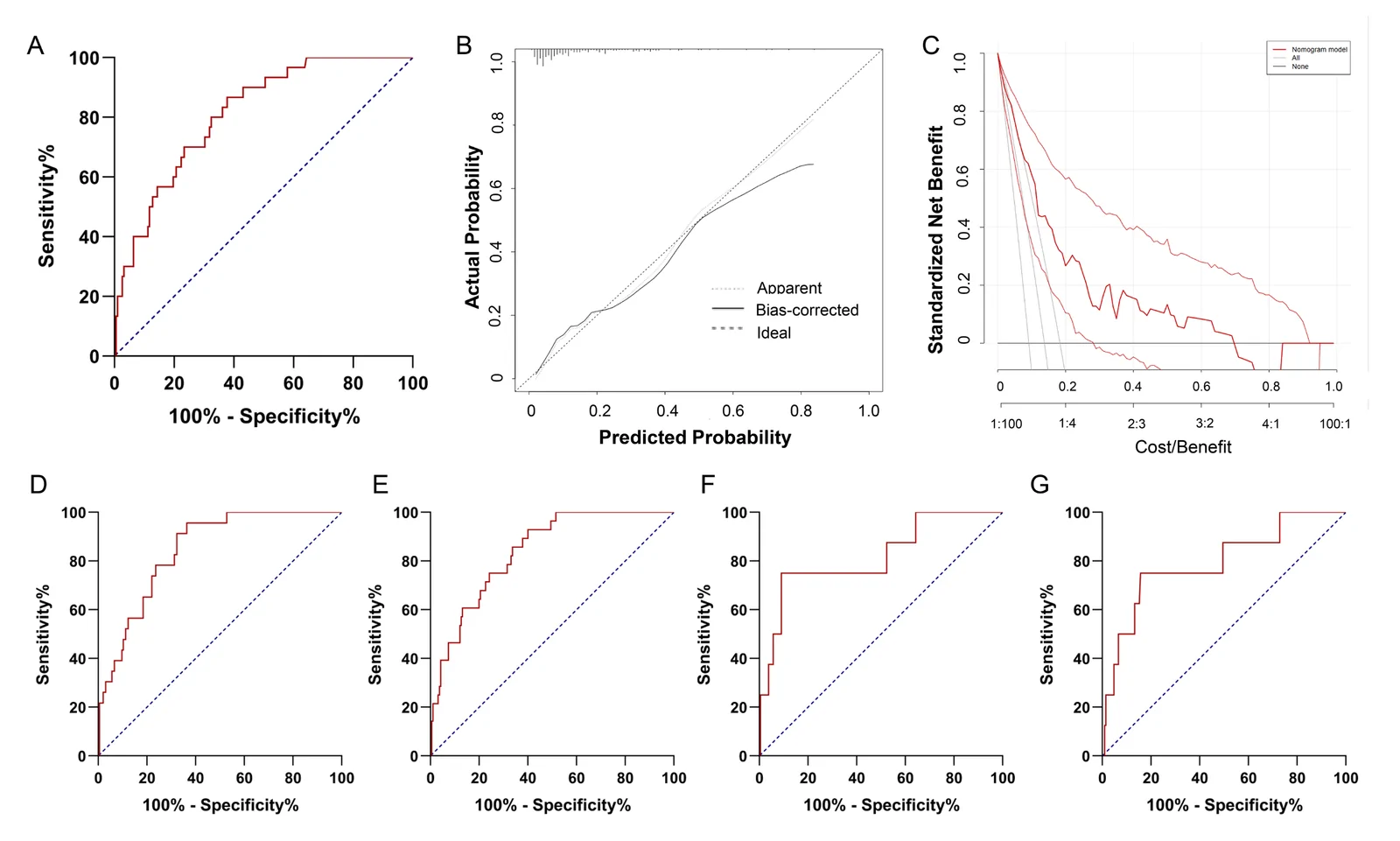
**The discriminatory ability and calibration of the model**. (**A**) The ROC curve for predicting SHS (AUC=0.812). (**B**) The calibration curve shows good agreement between predicted and observed probabilities (Hosmer-Lemeshow test P=0.855 and Brier score=0.098). (**C**) The DCA curve shows the model maximized clinical net benefits. ROC curves for predicting (D) cardiac dysfunction (AUC=0.846), (E) SHS excluding mild arrhythmia (AUC=0.838), (F) acute coronary syndrome (AUC= 0.818), (G) arrhythmia (AUC= 0.794).

A nomogram for SHS post-EVT was constructed based on the above four significant factors (Fig. 4). The predictive capacity of this model based on AUC reaches 0.812 (95% CI 0.738-0.886) (Fig. 5A). The calibration curve exhibits a strong alignment with the ideal diagonal line, suggesting a congruency between the predicted probability and the actual observed results (Fig. 5B). Furthermore, Hosmer-Lemeshow test (χ^2^ = 4.019, P = 0.855) and Brier score (0.098) indicate good calibration and conformity of the model. The DCA curve shows that the SHS prediction model maximized clinical net benefits for patients undergoing EVT (Fig. 5C). Meanwhile, internal validation exhibits a similar AUC (0.811, 95% CI 0.809-0.813), proving the robustness and reliability of the model.

### Sensitivity analysis

To evaluate the contribution of this nomogram to SHS subtypes and ensure its robustness, we performed subgroup analyses separately in patients with ACS (n=8), arrhythmia (n=8), cardiac dysfunction (n=23), and SHS excluding mild arrhythmia (n=28). The results demonstrate that the model exhibits the strongest predictive performance for cardiac dysfunction (AUC = 0.846, 95% CI 0.779-0.913, Fig. 5D), followed by SHS excluding mild arrhythmia cases (AUC = 0.838, 95% CI 0.771-0.905, Fig. 5E) and ACS (AUC = 0.818, 95% CI 0.642-0.995, Fig. 5F), while showing relatively weaker predictive efficacy for arrhythmia (AUC = 0.794, 95% CI 0.609-0.978, Fig. 5G). Moreover, these findings further indicate the robustness of the predictive model.

### Neurofunctional prognosis

**Figure 6.**
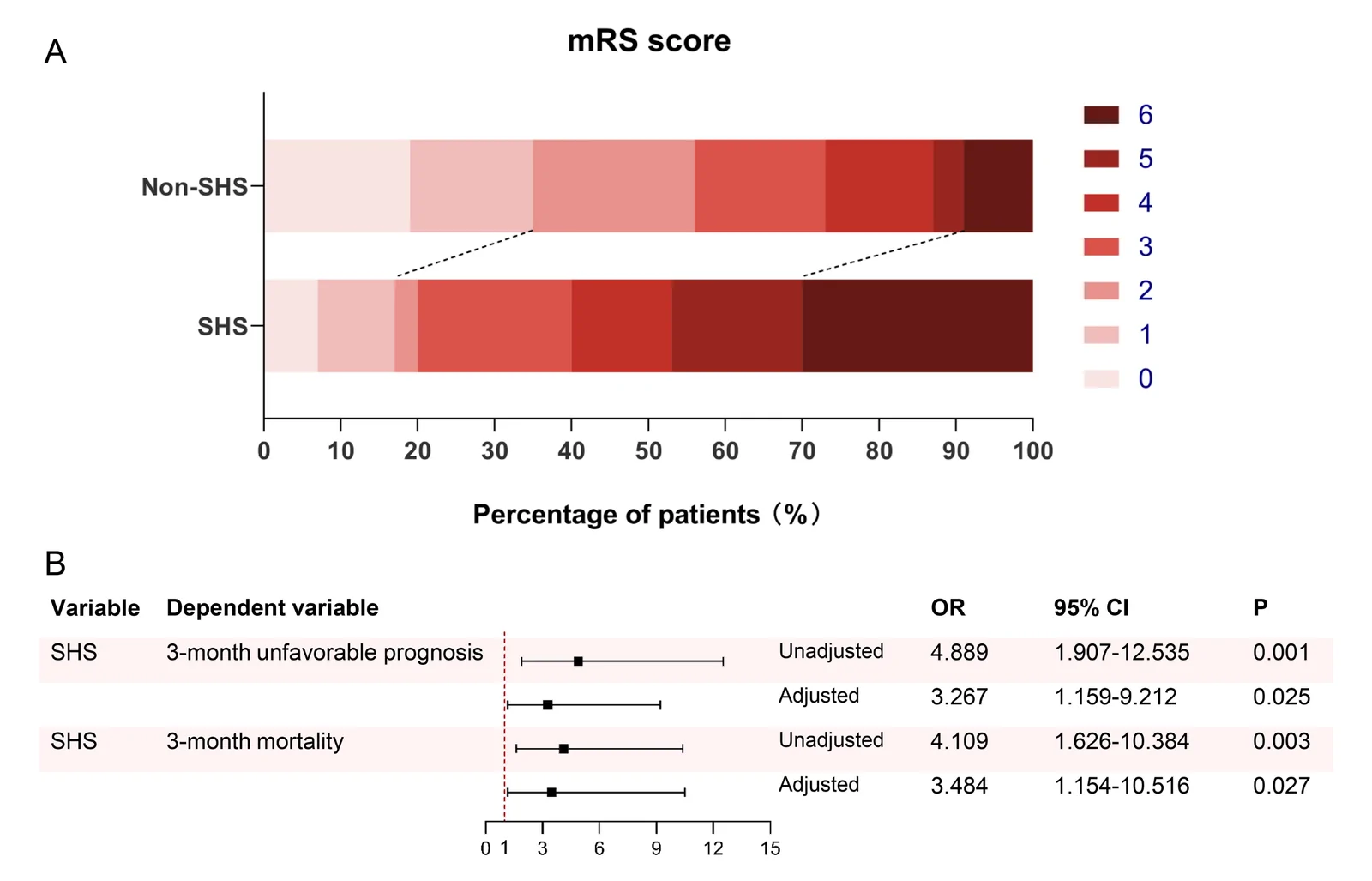
**Correlation between SHS and 3-month prognosis**. (**A**) Distribution of mRS scores of SHS group and non-SHS group. (**B**) Univariable and multivariable logistic regression analysis with 3-month unfavorable prognosis or mortality as dependent variable. Age, gender, BMI, and vascular risk factors were adjusted for multivariable logistic regression analysis.

The 3-month mRS scores of patients were obtained, with a total of 210 patients incorporated into the analysis after excluding 8 patients who were lost to follow-up. The mRS distribution is presented in Figure 6A. Statistical analysis reveals that 80.0% of patients in the SHS group experienced an unfavorable prognosis 3 months after stroke, which was notably higher than the non-SHS group (80.0% vs. 45.0%, P < 0.001). The risk of mortality within 3 months was substantially elevated among patients with SHS (30.0% vs. 9.4%, P = 0.004).

To clarify the correlation between SHS and prognosis in AIS patients undergoing EVT, logistic regression analyses were performed, with SHS serving as the variable and unfavorable prognosis/mortality as the dependent variable. The results reveal that SHS is an independent risk factor for 3-month unfavorable prognosis (OR 3.267, 95% CI 1.159-9.212, P = 0.025) and mortality (OR 3.484, 95% CI 1.154-10.516, P = 0.027) after adjusting for age, gender, body mass index (BMI), and vascular risk factors (Fig. 6B).

## DISCUSSION

This study investigated early SHS occurrence following EVT in patients with anterior circulation large vessel occlusion strokes, establishing a predictive model based on age, hyperlipidemia, creatinine, and TACI. This model fills an important gap in early SHS risk stratification for AIS patients undergoing EVT and has the potential to offer significant clinical benefits. Our analysis of 3-month functional outcomes further revealed that SHS occurrence was associated with worse neurological outcomes.

Data from previous studies indicate that 8.6%-17.0% of patients are readmitted for cardiac reasons within one month after EVT for AIS [21,22]. Due to the difficulty in obtaining patients’ pre-stroke cardiac conditions, determining the temporal sequence of cardiac changes and stroke onset poses a significant challenge. To minimize potential biases, our study excluded patients with a history of cardiac disease or stroke. We observed that 13.8% of AIS patients who underwent EVT developed cardiac complications within the first two weeks after stroke onset, with cardiac dysfunction representing the predominant manifestation, providing novel epidemiological evidence for early SHS following EVT. Given the retrospective nature of this study, some asymptomatic cardiac alterations, especially transient electrocardiographic abnormalities and changes in cardiac troponin levels, were not captured, suggesting that the actual incidence of SHS may be higher. Our findings indicated that SHS worsened prognosis and increased mortality in EVT patients at 3 months, underscoring the importance of an early prediction model.

Using LASSO regression and multivariate logistics regression analysis, four factors (age, hyperlipidemia, creatinine, and TACI) were included in the nomogram. This model demonstrated favorable predictive performance and robustness in SHS and its subtypes, particularly for cardiac dysfunction, SHS excluding mild arrhythmia, and ACS. Compared to existing scales, our model offered three distinct clinical advantages: (1) its EVT-specific design provided enhanced accuracy for this targeted population; (2) it maintained excellent diagnostic performance while utilizing fewer predictive variables, facilitating clinical implementation; and (3) it demonstrated high predictive value for SHS subtypes, including cardiac dysfunction, ACS, and arrhythmia [12].

Advanced age, an established independent risk factor for cardio-cerebrovascular disease, is significantly associated with post-AIS cardiac dysfunction and myocardial injury [23,24]. Aging progressively diminishes vascular elasticity and accelerates atherosclerotic changes. Concurrently, elderly individuals have higher burdens of conventional vascular risk factors and chronic diseases, collectively elevating their susceptibility to cardiovascular events [25]. Additionally, age-related immunosenescence increases vulnerability to SHS in elderly individuals, underscoring the necessity for rigorous monitoring and early prevention [26,27].

Hyperlipidemia significantly contributes to SHS development in EVT patients by prompting fatty deposition in vascular walls, exacerbating atherosclerosis, and increasing cardiovascular risk [28]. It further impairs myocardial function, compromising both systolic contractility and diastolic relaxation [29,30]. Simultaneously, hyperlipidemia can damage endothelial cells, triggering inflammatory responses—a pivotal mechanism underlying SHS pathogenesis [2].

TACI, characterized by complete infarction of the middle cerebral artery supply area, is associated with high NIHSS score, an established predictor of SHS [9,31]. It causes extensive blood-brain barrier disruption, promoting the spread of inflammatory cytokines and amplifying systemic inflammation [3]. Additionally, damaged blood-brain barrier allows brain-derived antigens and extracellular vesicles to enter the blood circulation, thereby exacerbating cardiac damage [32]. TACI frequently involves the insular cortex, triggering autonomic neural network disorders and catecholamine surges, which collectively contribute to cardiac structural and functional changes [2,33]. Previous studies have also provided evidence that insular damage (especially right insula) is closely related to the development of SHS [34]. TACI’s combination of high NIHSS and insular involvement provides a stronger predictive power for SHS compared to either factor alone.

Creatinine levels serve as a biomarker of renal function and are mechanistically linked to SHS pathophysiology through their associations with inflammatory cascades, oxidative stress responses, and endothelial dysfunction [35,36]. Even slight elevations in creatinine levels may heighten cardiovascular risk, potentially mediated through apolipoprotein A-I dysfunction-induced inflammatory pathways [37,38]. In addition, high creatinine levels have also been proven to make patients more susceptible to heart failure [39]. Previous studies found that higher creatinine levels were associated with cardiac damage after AIS, consisting with our results [4,40]. Notably, our study revealed a stronger univariate association between urea and SHS. However, statistical analysis showed that creatinine, when combined with other indicators, offered better predictive performance. Consequently, our model optimization process simultaneously maximized predictive accuracy and preserved model parsimony, resulting in the current final predictive model.

Beyond the established predictors incorporated in our risk model, a noteworthy finding was the significantly higher prevalence of female gender among SHS patients. A few studies have observed this phenomenon as well [41,42]. Existing evidence indicates that postmenopausal women exhibit heightened susceptibility to Takotsubo syndrome, potentially mediated by estrogen deficiency-induced endothelial dysfunction [43,44]. Although gender was not an independent risk factor for SHS after adjusting for age in our study, the potential role of hormonal factors in modulating the brain-heart axis following AIS merits particular research attention. Furthermore, female AIS patients demonstrate greater vulnerability to acute myocardial injury, despite exhibiting a much lower burden of atherosclerotic coronary artery disease compared to males [45,46]. This paradox suggests that cardiac injury in females may involve more acute, non-atherosclerotic mechanisms [47]. The pathophysiological mechanisms underlying gender differences in SHS warrants further exploration.

Finally, an innovative study suggests that intravenous thrombolysis prior to EVT may confer cardioprotective effects in AIS [19]. The proposed mechanism involves recombinant tissue plasminogen activator-mediated improvement of cardiac microcirculatory endothelial dysfunction, which is frequently impaired post-AIS [19,48]. However, our analysis revealed no statistically significant difference in SHS incidence between patients treated with bridging EVT and those receiving direct EVT. In addition, this study did not identify significant associations between SHS and other interventions including general anesthesia, intra-arterial thrombolysis, tirofiban administration, thrombus aspiration, stent thrombectomy, stent implantation, or surgery. However, these findings may reflect limited statistical power due to the modest sample size rather than true irrelevance. Future prospective studies employing treatment-stratified designs with adequate power are warranted to definitively evaluate these potential associations.

In summary, this nomogram provided early prognostic risk stratification for AIS patients undergoing EVT by incorporating a concise panel of easily accessible clinical parameters: age, hyperlipidemia, TACI, and creatinine. During the initial EVT assessment workflow, neurologists can utilize information obtained from standard preoperative evaluations—including laboratory tests and neuroimaging findings—to generate individualized risk scores via the nomogram. The resulting risk stratification not only aids in clinical decision-making but also holds significant potential for optimizing post-EVT management. For early cardiac monitoring, the model facilitates identification of high-risk patients susceptible to hemodynamic instability or arrhythmia, enabling targeted interventions. Patients with elevated risk scores may particularly benefit from extended cardiac monitoring post-EVT, allowing timely detection of silent arrhythmia or ischemic cardiac events. Regarding preventive strategies, the model provides valuable guidance for personalized treatment plans. For instance, clinicians may optimize the frequency of follow-up cardiac evaluations (e.g., echocardiography, electrocardiography, coronary angiography) for high-risk patients to improve clinical outcomes and resource allocation. Further prospective validation studies are warranted to quantify the model's clinical impact.

However, our study had several limitations. First, the retrospective nature of this study limited our ability to identify asymptomatic or transient cardiac events, potentially introducing bias. Implementation of continuous electrocardiogram monitoring and serial troponin assessments in future prospective studies could enhance endpoint detection and strengthen the model's clinical applicability. Second, while excluding patients with pre-existing cardiac conditions enhanced the specificity of SHS assessment, this may limit transportability to real-world settings. Future prospective studies should incorporate subgroup analyses stratified by pre-existing cardiac conditions to enhance the model's generalizability across diverse clinical populations. Third, the single-center design with limited sample size and no external validation may affect the broader applicability of our results. Future validation through larger-scale, multicenter studies will be crucial to confirm the model's clinical impact, robustness, and applicability.

### Conclusions

SHS significantly increases the risk of unfavorable outcomes and mortality in AIS patients treated with EVT. The nomogram, which incorporates age, hyperlipidemia, TACI, and creatinine levels, exhibits robust accuracy for early SHS detection. While this model shows significant potential for guiding early clinical decision-making and improving prognosis, its generalizability necessitates validation in large-scale, multicenter studies.
